# 7-Methoxy-1-Tetralone Induces Apoptosis, Suppresses Cell Proliferation and Migration in Hepatocellular Carcinoma via Regulating c-Met, p-AKT, NF-κB, MMP2, and MMP9 Expression

**DOI:** 10.3389/fonc.2020.00058

**Published:** 2020-02-07

**Authors:** Ying Wen, Xiaoyan Cai, Shaolian Chen, Wei Fu, Dong Chai, Huainian Zhang, Yongli Zhang

**Affiliations:** ^1^Guangzhou Key Laboratory of Construction and Application of New Drug Screening Model Systems, Guangdong Pharmaceutical University, Guangzhou, China; ^2^Department of Cell Biology and Medical Genetics, School of Life Sciences and Biopharmaceutics, Guangdong Pharmaceutical University, Guangzhou, China; ^3^Department of Clinical Laboratory, The First Affiliated Hospital, Guangdong Pharmaceutical University, Guangzhou, China

**Keywords:** 7-methoxy-1-tetralone, hepatocellular carcinoma, cell proliferation, cell apoptosis, cell migration

## Abstract

This study aimed to determine the anti-proliferative and anti-migratory effects of 7-methoxy-1-tetralone (MT) in hepatocellular carcinoma (HCC) cells. MTT assay assessed HCC cell viability; cell apoptosis of HCC cells was determined by flow cytometry; wound healing assay evaluated HCC cell migratory ability; protein expression levels were assessed using western blot assay; the *in vivo* antitumor effects of MT were tested in BALB/c nude mice and the pathological changes within the tumor tissues were evaluated by immunohistochemistry. MT treatment significantly suppressed the cell proliferative and migratory potentials of HepG2 cells, and induced HepG2 cell apoptosis. The western blot assay showed that MT treatment caused a suppression on c-Met, phosphorylated AKT (p-AKT), NF-κB, matrix metallopeptidase 2 (MMP2)/MMP9 protein levels in HepG2 cells. Further *in vivo* animal studies deciphered that MT treatment suppressed tumor growth of HepG2 cells in the nude mice, but had no effect on the body weight and the organ index of liver and spleen. Further immunohistochemistry analysis of the dissected tumor tissues showed that MT treatment significantly suppressed the protein expression levels of NF-κB, MMP9, MMP2, and p-AKT. In summary, the present study demonstrated the anti-tumor effects of MT on the HCC, and MT suppressed HCC progression possibly via regulating proliferation- and migration-related mediators including c-Met, p-AKT, NF-κB, MMP2, and MMP9 in HepG2 cells.

## Introduction

Hepatocellular carcinoma (HCC) represents one of the most severe human malignancies with a high mortality (1). High risk factors such as hepatitis B virus (HBV) or hepatitis C virus (HCV) infection, alcohol abuse, hemochromatosis psychosis, and so on, may lead to a chronic liver disease, then with progression to cirrhosis, eventually leading to the occurrence of HCC (2–4). Surgical interventions (surgical resection, liver transplantation, and locoregional therapies) have been playing important roles in the treatment of HCC (5, 6). However, only 15% patients are eligible for the potentially curative treatments since a majority of patients diagnosed with HCC already have liver dysfunction and/or are at advanced stages (III or IV), and these patients cannot benefit from these interventions (7, 8). Sorafenib is the only FDA-approved medication for the management of advanced HCC (9, 10). However, a large percentage of patients still experience disease progression after sorafenib treatment (11). Therefore, there is still a large unmet medical need to develop new therapeutic strategies to treat HCC.

7-methoxy-1-tetralone (MT) is mainly used as the organic material or intermediate in chemical engineering currently (12–15). Our research group revealed that wild Juglansshurica leather has strong anti-tumor effects through the long-term study, while MT may be one of the effective components. Sun et al. (16) revealed that the compounds extracted from green peel of *Juglans mandshurica* possessed the insecticidal activities, further investigation deciphered that MT is one of the major active components (the relative content: 6.81%). Recently, studies showed that extracts from green peel of *J. mandshurica* exhibited moderate inhibitory effects on the lung cancer cells (17) Nevertheless, systematic study of MT's potential to repress human hepatoma cell growth has not been documented.

This study was undertaken to gain deeper insights into the anti-hepatocellular carcinoma activities and anti-neoplastic molecular mechanisms of MT. Changes to cell proliferation, apoptosis and migration and AKT, phosphorylated AKT (p-AKT), NF-κB, and matrix metallopeptidase 2 (MMP2)/MMP9 protein expression following application of MT are defined in this study using *in vitro* cell culture and *in vivo* animal experiments, in order to provide the experimental basis for its future clinical application.

## Materials and Methods

### Cell Culture and Chemical Reagents

The two human hepatoma cell lines (HepG2 and LO2) were a generous gift from Sun Yat-sen University. HepG2 and LO2 cells were kept in DMEM (Thermo Fisher Scientific, Waltham, USA) supplemented with 10% fetal bovine serum (FBS; Thermo Fisher Scientific) in a humidified incubator (Sanyong, Tokyo, Japan) with 5% CO_2_ at 37°C. Dimethyl sulfoxide (DMSO) was used to dissolve MT (purity > 98%; Sigma-Aldrich, St. Louis, USA) to prepare the stock solution, and the stock solution was diluted with cell culture medium as the respective working concentrations, and the concentration of DMSO in the working solution was <0.1% (18).

### Cell Viability Assay

The anti-proliferative effects of MT were evaluated by MTT assay. LO2 and HepG2 (1 × 10^5^ cells/well) were seeded at 96-well plates. The seeded cells were subjected to incubate with different concentrations of MT (31.25, 62.5, 125, 250, 500, and 1,000 μM) for 24, 48, and 72 h, respectively. Fluorouracil (5-FU, 50 μM) served as a positive control. After 4 h incubation with MTT (5 mg/ml) at 37°C. Cell viability was evaluated by measuring the absorbance at 570 nm.

### Flow Cytometry Analysis of Cell Apoptosis

Apoptosis was determined using flow cytometer with a commercial Annexin V-FITC Apoptosis Detection Kit (KaiJi, Nanjing, China) by following the manufacture's protocol. In brief, HepG2 cells were subjected to treatment with different concentrations of drugs for 48 h after plating as a monolayer. Cells were rinsed twice with cold phosphate buffered saline (PBS) and trypsinized gently using the trypsin reagent, then cells were re-suspended in 1× binding buffer and were incubated FITC Annexin V and propidium iodide (PI) for 15 min at room temperature in the dark. A BD FACSCalibur flow cytometer (BD Biosciences, Franklin Lakes, USA) was used to identify the different subpopulations of apoptotic cells.

### Wound Healing Assay

HepG2 cells after different treatments were allowed to grow in 6-well plates until ~90% confluence. A sterile 200 μL pipette tip was used to create a wound in the HepG2 cell monolayer. HepG2 cells were rinsed twice with PBS to remove debris, and HepG2 cells were incubated with serum-free medium for indicated time durations. At indicated time points, images of the plates were acquired under a microscope and the migrating distances were analyzed by Image-Pro-Plus software (19).

### Western Blot Analysis

Total proteins were obtained by lysing the cells or tissues using RIPA buffer (Beyotime). The BCA quantitative analysis kit was used to measure concentrations of protein samples (Beyotime). Equal aliquots of protein samples were separated by sodium dodecyl sulfate-polyacrylamide gel electrophoresis and the proteins were electro-transferred onto a PVDF membrane (Millipore, Burlington, USA). The PVDF membrane was probed with different primary antibodies, and protein bands were visualized by an enhanced chemiluminescence method (Thermo Fisher Scientific). Antibodies for c-Met (Cat. #4560), Akt (Cat. #4685) p-Akt (Cat. #4060), NF-κB (Cat. #8242), MMP2 (Cat. #40944), MMP9 (Cat. #13667), β-actin (Cat. #8457) and the corresponding secondary antibodies were obtained from Cell Signal Technology Inc. (Danvers, USA).

### *In vivo* Animal Studies to Assess Tumor Growth

Five-week-old BALB/c nude mice (*n* = 25) used for *in vivo* experiments were purchased from the Guangdong Laboratory Animal Center. HepG2 cells (7 × 10^6^ cells/ml) were subcutaneously administered into the right sub-axillary region of the mice. When the diameters reached a length >3 mm, mice were randomly assigned to five groups and administered with different drugs by the intraperitoneal injection. Mice from the control group mice received saline, mice from the positive control group mice received 5-FU (15 mg/kg/d) and mice from the experimental group received MT (80, 120, or 160 mg/kg/d). Subcutaneous tumors were measured using calipers, and tumor volume was assessed using the following formula: width^2^ × length × 0.5 (20). Body weight was measured every day. Mice received 19 doses in total and were sacrificed for harvesting tumors at 24 h after the last dose. The organ index for liver and spleen was determined as organ weight (g)/body weight (g). All the experimental procedures of the animal studies were under the approval by the Animal Ethics Committee of Guangdong Pharmaceutical University.

### Hematoxylin and Eosin (HE) Staining Assay

HE staining assay was used to observe pathological changes within the tumor tissues. Tumor tissues were embedded in paraffin, sectioned into 4 μm slices and deparaffinized in xylene for 15 min followed by re-hydration in the graded ethanol. After staining with hematoxylin for 5–10 min, the slides were incubated with 1% hydrochloride alcohol for 20 s. Following 5–10 min running water washing, the slides were stained with 0.5% eosin for 3 min. The morphology of the HE-stained tumor tissues were evaluated using a light microscope.

### Immunohistochemical Analysis of Tumor Tissues

The paraffin sections were deparaffinized using xylene and hydrated through graded ethanol. After antigen retrieval, 3% H_2_O_2_ was used to quench endogenous peroxidase. After PBS washing, slides were incubated with 10% bovine serum albumin (BSA) for 10 min, and then primary antibodies including MMP2 (1:200), MMP9 (1:200), p-Akt (1:500) and NF-κB (1:100) were added to incubate with the slices overnight at 4°C. After that, slides were incubated with secondary antibodies for 30 min at room temperature. 3,3′- diaminobenzidine (DAB) as a chromogen was used to visualize the antigens. After counterstaining with hematoxylin, the slides were dehydrated and mounted for viewing. For the negative control, primary antibody was replaced by BSA. The percentage of positive staining area was measured by Image J.

### Statistical Analysis

A SPSS statistical software package (IBM, Armonk, USA) was used for performing data analysis. Results data are presented as the mean ± standard deviation. Differences between the mean values for the different groups were determined using one-way ANOVA followed by Dunnett's multiple comparison test. A *P*-value less than 0.05 was defined to be statistically significant.

## Results

### MT Inhibited Proliferation of HepG2 Cells but Has Little Cytotoxic Effects on Normal LO2 Cells

**Figure 1:**
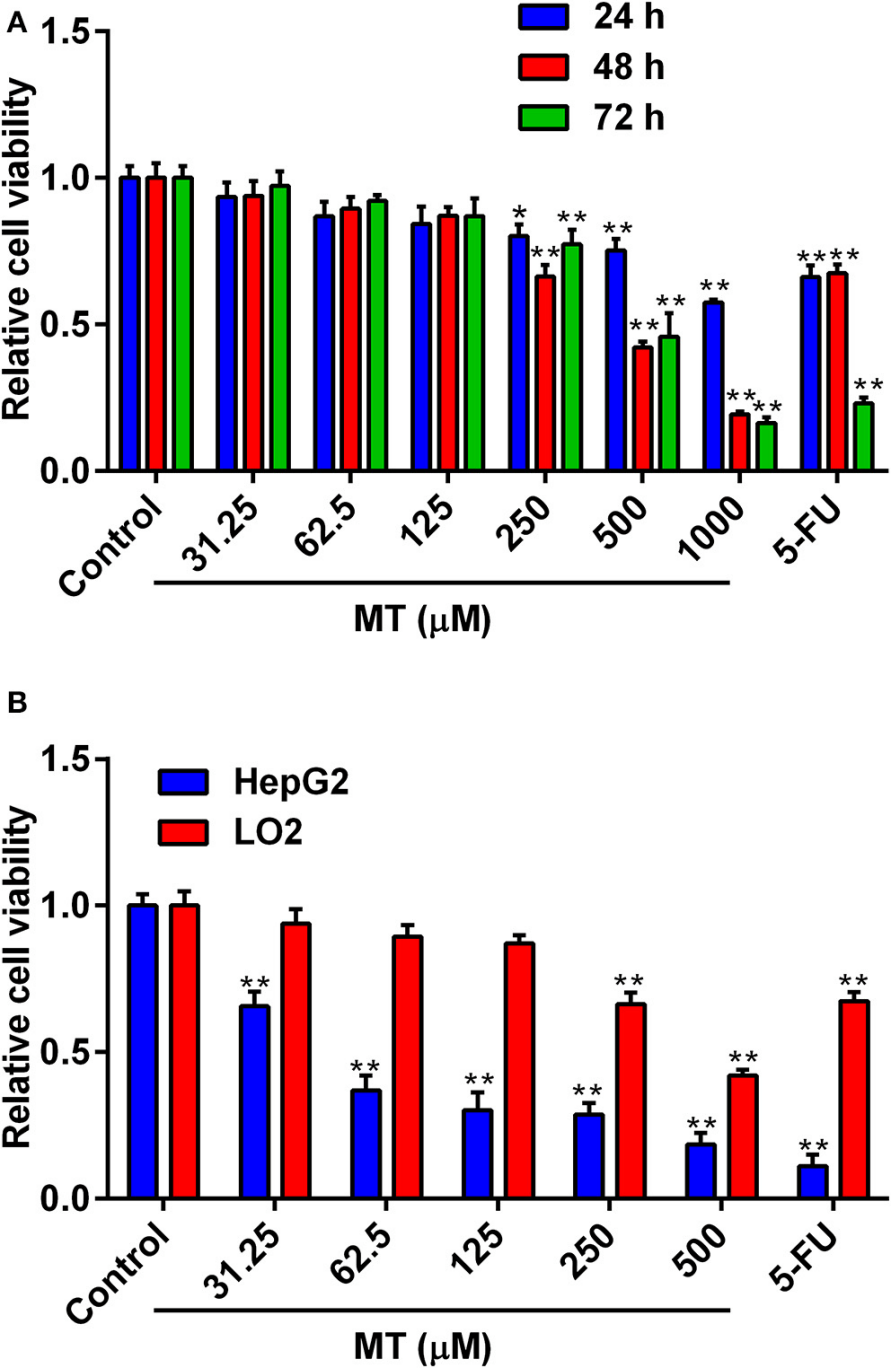
Anti-proliferative activity of MT on LO2 and HepG2 cells. **(A)** Effects of MT and 5-FU on the proliferation of LO2 cells were determined by MTT assay. **(B)** Effects of MT and 5-FU on the proliferation of HepG2 cells and LO2 cells at after 48 h treatment with MT were determined by MTT assay. Data from at least three independent experiments performed in triplicates are presented; *P < 0.05 and **P < 0.01 compared to the corresponding control groups.

The cytotoxic effects of MT on LO2 cells were determined by treating cells with different concentrations of MT (0–1,000 μM) for 24, 48, 72 h, respectively. In addition, HepG2 cells were subjected to incubate with elevated MT concentrations (0–500 μM) for 48 h. 5-Fluorouracil (5-Fu, 50 μM) was used as a positive control in this investigation. As shown in Figure 1A, increasing concentrations of MT exhibited anti-proliferative actions in LO2 cells in a time- and concentration-dependent way. Nevertheless, the 48 h treatment duration was selected for our follow-up study because MT had the greatest dose-dependent effect on LO2 cells at 48 h after treatment. The results in Figure 1B indicated that MT was more effective to inhibit the proliferation of HepG2 cells when compared with LO2 cells. For subsequent experiments three different concentrations for MT (40,100, 250 μM) were used.

### Effects of MT on the Cell Apoptosis of HepG2 Cells

**Figure 2:**
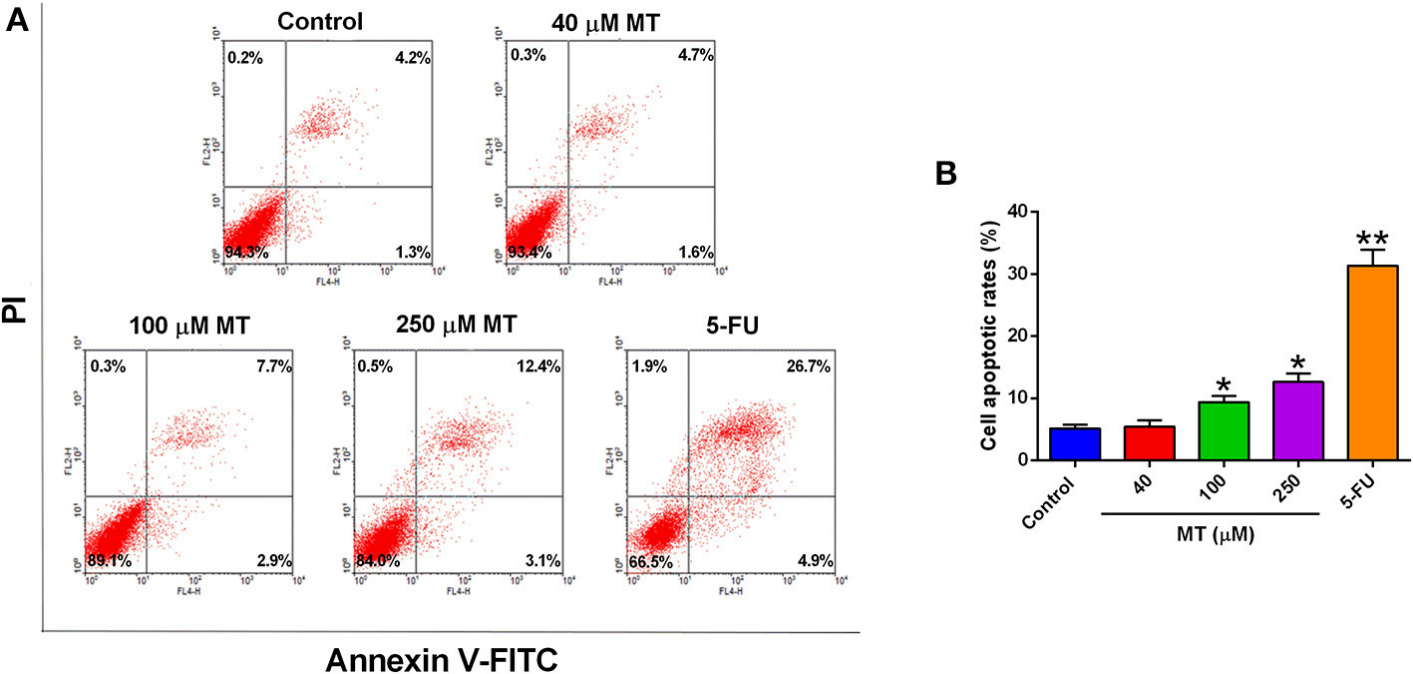
Effects of MT treatment on the cell apoptosis of HepG2 cells. **(A)** HepG2 cells were incubated with different concentrations of MT for 48 h and analyzed by flow cytometry. Histograms showed that distribution of apoptotic cells after different treatment. **(B)** Quantification of cell apoptosis from the histograms. Data from at least three independent experiments performed in triplicates are presented; *P < 0.05 and **P < 0.01 compared to the corresponding control groups.

To validate the effects of MT on HepG2 cell apoptosis, flow cytometry was performed to analyze Annexin V-FITC/PI stained HepG2 cells. MT treatment mildly increased the number of apoptotic cells (AV+PI+ and AV+PI- cells) compared to the control group (Figure 2). The percentage of apoptotic HepG2 cells following treatment with 0, 40, 100, or 250 μM MT for 48 h were 5.44 ± 0.84, 5.07 ± 1.22, 7.15 ±1.92, and 11.45 ±1.11%, respectively (Figure 2). On the other hand, 5-FU induced much more HepG2 apoptotic cells than that treated with MT (Figure 2).

### MT Suppressed Migration in HepG2 Cells

**Figure 3:**
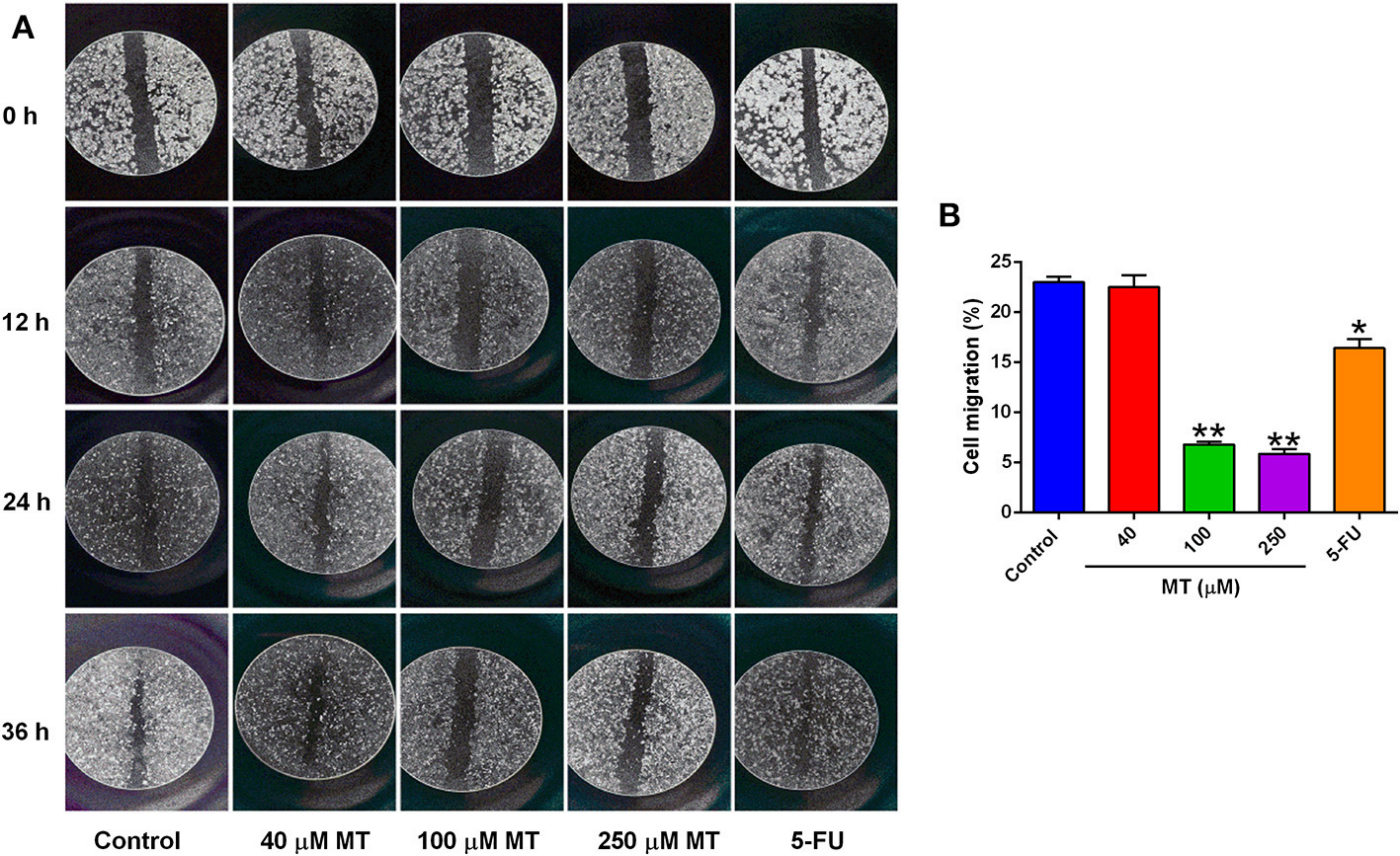
Effects of MT on HepG2 cell migration was determined by wound-healing test. **(A)** Representative images (at a magnification of 5×) of the wound healing of HepG2 cells after different drug treatments. **(B)** Quantification of cell migration of HepG2 cells after different treatments. Data from at least three independent experiments performed in triplicates are presented; *P < 0.05 and **P < 0.01 compared to the corresponding control groups.

Wound healing assay was undertaken to evaluate the effect of MT on HepG2 cell migration. As demonstrated in Figure 3, the cells of different concentration group were tightly connected at 0 h. The scratch of the control group, 40 μM low dose group and 5-FU group was significantly prolonged at 12, 24, and 36 h after drug treatment, while 100 and 250 μM groups were not obvious, indicated that the healing of the scratch was significantly reduced following treatment with 100 and 250 μM MT (Figure 3).

### Effects of MT on c-Met, NF-κB, p-Akt, Akt, and MMP2/MMP9 Protein Expression in HepG2 Cells

**Figure 4:**
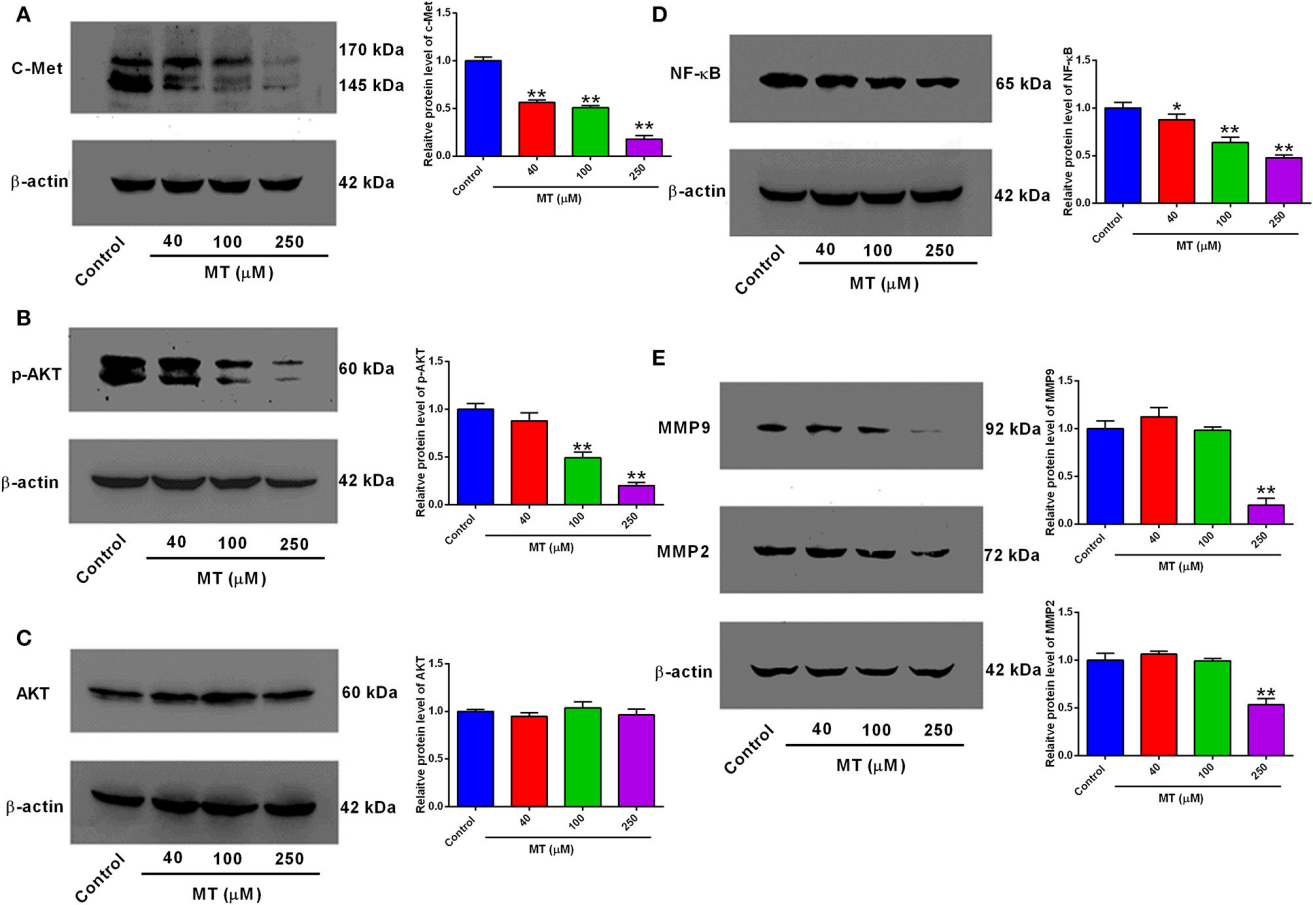
Effects of MT treatment on the protein expression levels of c-Met, p-AKT, AKT, NF-κB, MMP2, and MMP9 in HepG2 cells. **(A)** c-Met, **(B)** p-AKT, **(C)** AKT, **(D)** NF-κB, **(E)** MMP9 and MMP2 in MT-treated HepG2 cells were assessed by Western Blot analysis and were quantified using ImageJ software. Data from at least three independent experiments performed in triplicates are presented; *P < 0.05 and **P < 0.01 compared to the corresponding control groups.

**Figure 5:**
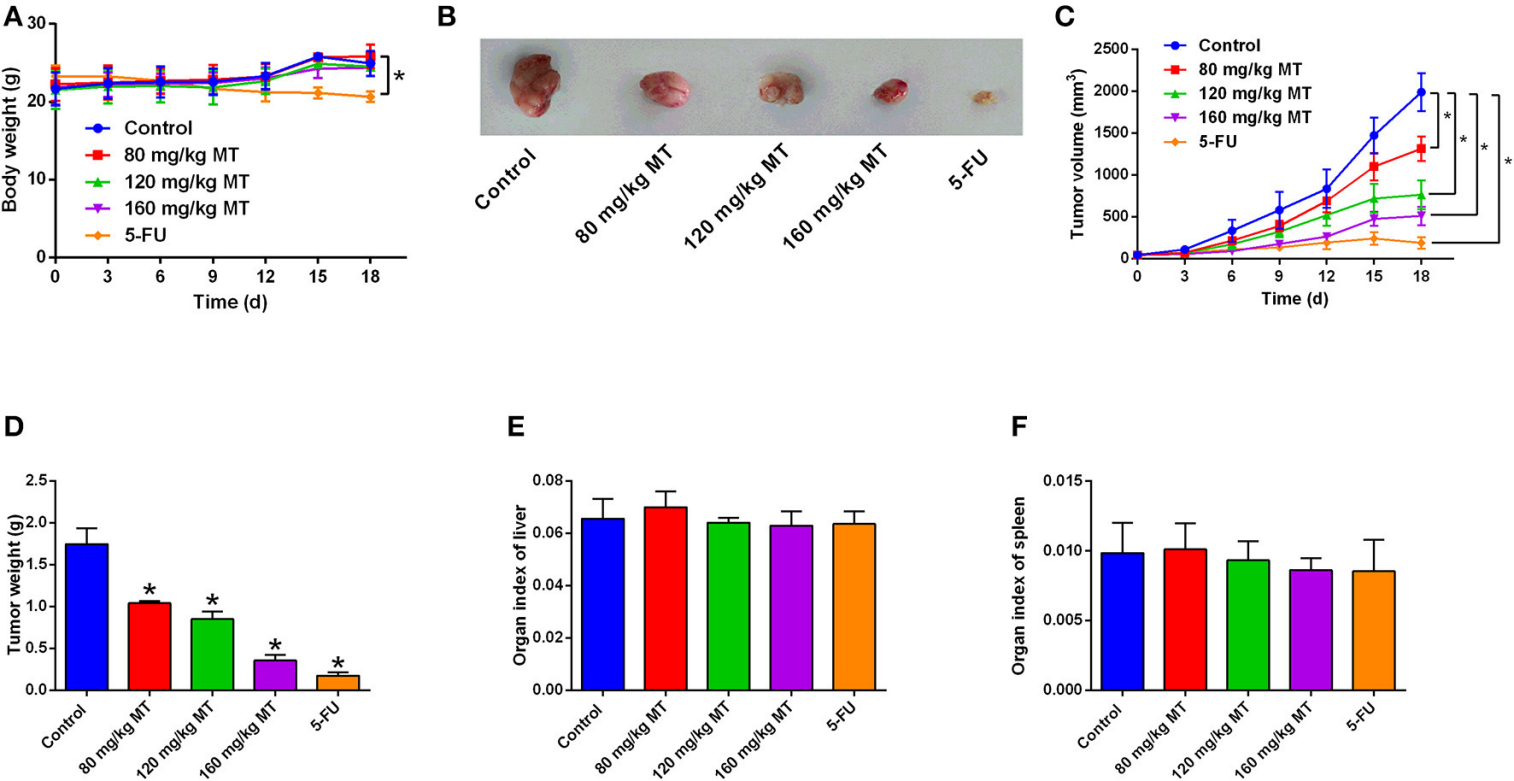
MT inhibits tumor growth *in vivo*. **(A)** Body weight of mice after different treatments were determined every day. **(B)** Representative images of tumor tissues dissected from mice after different treatments. **(C)** The tumor volumes were measured every 3 days for 19 days in different treatment groups. **(D)** The tumor weight was measured after all nude mice were sacrificed. **(E,F)** Organ index of liver **(E)** and spleen **(F)** in the nude mice after different treatments were determined. *N* = 5. *P < 0.05 compared to the corresponding control groups.

To assess the significance of the expression patterns of proliferation and migration-related proteins in response to MT, HepG2 cells were subjected to treatment with elevated MT concentrations (0, 40, 100, and 250 μM) for 48 h, and expression levels of c-Met, AKT, p-AKT, NF-κB, and MMP2/9, which closely involved in the regulation of proliferation and migration were demonstrated in Figure 4. The expression of c-Met, p-Akt, NF-κB, and MMP2/9 significantly decreased compared with control group after 48 h of treatment with HepG2 cells with 40, 100, and 250 μM MT (Figure 5B), while the expression change of Akt protein was not obvious. MT at concentration of 40 μM significantly repressed c-Met and NF-κB protein expression levels (Figure 4); MT at a concentration of 100 μM significantly down-regulated p-AKT, NF-κB protein expression (Figure 4); MT at a concentration of 250 μM inhibited c-Met, p-AKT, NF-κB, and MMP2/MMP9 protein expression levels (Figure 4); while the AKT protein levels were not affected by MT treatment in HepG2 cells (Figure 4).

### MT Inhibits Tumor Growth *in vivo*

BALB/c nude mice were sacrificed after 19 days receiving MT, 5-FU, or saline by intraperitoneal injection. MT treatment failed to affect mice body weight, while 5-FU treatment reduce the mice body weight (Figure 5A). The photos of representative tumor images from different treatment groups are demonstrated in Figure 5B. Tumor volume and tumor weight were significantly reduced by MT and 5-FU treatment when compared to the control group (Figures 5C,D). The tumor inhibition rates were 40.57% (80 mg/kg MT group), 51.43% (120 mg/kg MT group), 79.43% (160 mg/kg MT group), and 89.71% (5-FU group). MT and 5-FU had no effects on the organ indexes of liver or spleen (Figures 5E,F).

### Pathological Morphology and Relative Protein Expressions *in vivo*

**Figure 6:**
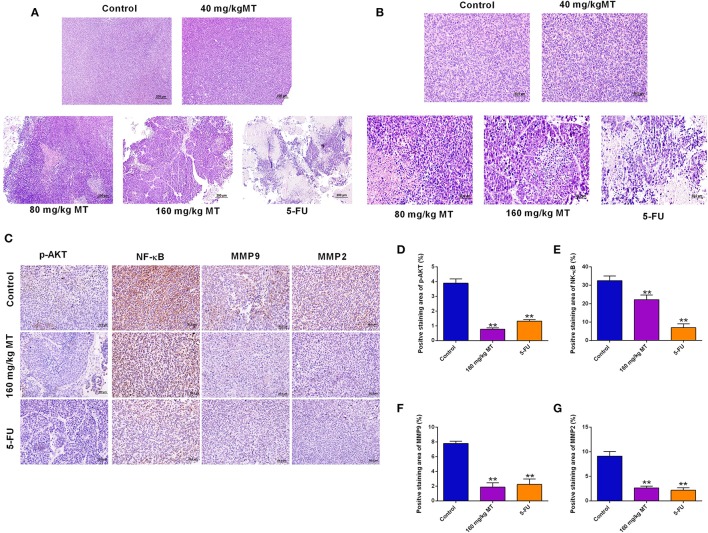
Pathological morphology and relative protein expressions in the tumor tissues. **(A,B)** HE staining of tumor of nude mice from different treatment groups, original magnification: **(A)** 10× **(B)** 40×. **(C)** Immunohistochemistry for p-Akt, NF-κB, MMP9, and MMP2 in tumors of nude mice in different groups, original magnification: 40×. **(D–G)** The results of the ratio of positive staining of p-Akt **(D)**, NF-κB **(E)**, MMP9 **(F)** and MMP2 **(G)** are analyzed and Quantified by ImageJ. *N* = 5. **P < 0.01 compared to the corresponding control groups.

Pathological morphology of tumor tissues was detected in saline group, MT (80, 120, 160 mg/kg) groups and 5-FU group and were presented in Figures 6A,B. In the saline control group, tumor cells were mainly intact. While, in the MT and 5-FU group, some of tumor cells in the exposed area were characterized with necrosis and cell fragmentation. These observations suggested that the cells were in a vigorous growth stage. However, in different dose of MT and 5-Fu groups, the typical increased nuclei volume and shrunken intercellular space were considerably reduced, and some chromosome fractures and integrated nuclear membranes were observed. Immunohistochemistry staining (Figure 6C) presents the four proteins (p-Akt, NF- κB, MMP9, and MMP2) expressions in saline group, 160 mg/ml MT group and 5-FU group, respectively. The percent of positive staining in saline group are: p-Akt (2.91 ± 0.96%), NF-κB (35.47 ± 1.78%), MMP9 (7.64 ± 1.27%), and MMP2 (9.69 ± 1.34%). The positive staining ratio of the four proteins in MT (160 mg/kg) group were all significantly lower; the positive expression of the four proteins in 5-FU group was also remarkably repressed (Figures 6D–G).

## Discussion

In the current investigation, we demonstrated the antitumor effects of MT against HCC progression and deciphered the underlying molecular mechanisms. Our results illustrated that MT treatment concentration-dependently repressed HCC cell viability. Mechanistically, MT downregulated NF-κB, Akt, p-Akt, MMP2/MMP9 expression levels in HCC cells. This inhibitory effect was further verified in a BALB/c nude mouse tumor-hypodermic transplantation model. Thus, MT requires further assessment as an effective strategy for the HCC treatment.

HCC metastasis largely contributed to the recurrence of this human malignancy (21). The key process for tumor metastases involves the dissolved surrounding tumor matrix and basement membrane caused by tumor-associated proteases (22). Therefore, MTT assay and wound healing assay was carried out to detect the HepG2 cell growth and migratory abilities, respectively, after treatment with MT in our study. Our results elucidated that MT remarkably repressed the proliferation and migration of HepG2.

Potential molecular mediators that were involved in the inhibitory actions of MT on HepG2 cell proliferation and migration were analyzed. MMPs has been well-documented for their regulatory actions on tumor metastasis. Studies found that dysregulated MMP2/9 in the solid tumors largely contributed to the tumor metastasis including HCC (23, 24). Additionally, the cell growth inhibitory effect could also attributed to the inhibition of MMPs (25). C-Met is a high-affinity receptor and can be targeted activated by hepatocyte growth factor (HGF). The activation of c-Met involves various signaling pathways including PI3K/Akt and MAPK/Erk signaling pathways, which are key mediators in HCC cell proliferation and metastasis (26–28). Moreover, studies also illustrated that deregulated c-Met is associated with the invasiveness and progression of HCC (29–31) and anti-tumor mechanisms of many components extracted from Chinese herbal medicine and plants were related to the expression level of HGF/c-Met (31–38). MT showed moderate enhancing effects on the HepG2 cells apoptosis, but remarkably inhibited the migratory potential of HepG2 cells, suggesting that the inhibitory actions of MT may be more relevant to the tumor metastasis. In this study, we further investigated into the PI3K/AKT/NF-κB signaling pathway, which represents a key pathway in regulating HCC progression. Activation of PI3K/AKT/NF-κB signaling pathway is effective to enhance HCC cell proliferative, invasive and migratory potentials. Activation of NF-κB requires the Akt phosphorylation, which stimulates the IκB kinase complex, phosphorylates and inactivates IκB (39–42). There is evidence showing that NF-κB up-regulated MMP-9 expression (43–45), while the NF-κB inhibition could downregulate MMP-2 expression (46). In current investigation, MT treatment remarkably repressed Akt phosphorylation and NF-κB activation, which may lead to the suppressed MMP2/9 expression in HepG2 cells. Collectively, our results revealed a potential mechanism of MT-mediated tumor-suppressive actions in HCC.

From a clinical perspective, evaluation of chemotherapeutics should consider their antitumor effects as well as financial cost and adverse effects. In the current investigation, we revealed that MT did not affect mice body weight in comparison with 5-Fu treatment, which may suggest the potential application of MT in antitumor therapy with limited adverse effects. In addition, our data implied that MT treatment of HCC may be acted via repressing c-Met/PI3K/AKT pathway, thereby inhibited MMP2/9.

There are several study limitations for the current work. MT induced moderate apoptosis in the HepG2 cells, and the molecular mechanisms underlying MT-mediated HCC cell apoptosis require further examination. The anti-tumor effects of MT were determined in one HCC cell line, and other types of HCC cell lines should be employed to confirm the anti-tumor effects of HCC. The expression levels of p-AKT, NF-κB, MMP9/2 were only quantified by IHC in the tumor tissues, and further studies may employ western blot assay to determine the levels of these proteins in the tumor tissues.

In summary, the present study demonstrated the anti-tumor effects of MT on the HCC, and MT suppressed HCC progression possibly via regulating proliferation and migration-related mediators including c-Met, p-AKT, NF-κB, MMP2, and MMP9 in HepG2 cells. However, further examination is required to assess the therapeutic potential of MT in HCC.

## Data Availability Statement

The raw data supporting the conclusions of this article will be made available by the authors, without undue reservation, to any qualified researcher.

## Ethics Statement

The animal study was reviewed and approved by Animal Ethics Committee of Guangdong Pharmaceutical University.

## Author Contributions

YZ and YW designed the study and wrote the manuscript. YW, XC, and SC performed the experiments and collected data. WF and DC performed statistical analysis. HZ performed the animal studies. All authors read and revised the manuscript before submission.

### Conflict of Interest

The authors declare that the research was conducted in the absence of any commercial or financial relationships that could be construed as a potential conflict of interest.
